# P349: Contribution of the private health sector to improve the performance of health systems in Africa

**DOI:** 10.1186/2047-2994-2-S1-P349

**Published:** 2013-06-20

**Authors:** J Boguifo

**Affiliations:** 1 Association of Private Clinics in Côte d'Ivoire (ACPCI) - EBRD SA, Abidjan, Côte d'Ivoire

## Objectives

The private hospital sector has an important role to play in the health systems of African countries. This sector remains, however, outside national strategies for health development.

## Methods

The Public Private Partnership (PPP) is an instrument that offers the opportunity to contribute more effectively to the performance of health systems.

## Results

The example shown is that of Côte d'Ivoire, where the private sector represents about 40% of the national supply of care. It counts 1,200 physicians and more than 1,000 hospital beds out of a total of about 3000. Private health institutions (ESP) generate many jobs and contribute significantly to the national GDP. It is nevertheless made of particular enterprises, which unlike the others, are subject to a double logic, without the benefit as it should, or incentives (tax, VAT, access to the PSP), or subsidies.

The public-private partnership (PPP) is an effective tool to develop between the ministries in charge of health and all players in the private health sector in African countries, particularly with the recurrent crises on the continent the issues related to the accessibility, quality and safety of care are fundamental today.

## Conclusion

In this context, the development perspectives of private hospitalization in Africa are in three main directions: Generalization of private clinics implementing a quality system for certification and even accreditation; Capacity building of health personnel, and institutional capacities in terms of infrastructure and technical facilities and Building bridges between the health systems of African countries, with regard to the PPP, collection and processing of medical data, sharing experiences.

## Disclosure of interest

None declared

